# AIM: A Mapping Program for Infrared Spectroscopy of Proteins

**DOI:** 10.1021/acs.jctc.2c00113

**Published:** 2022-04-07

**Authors:** Kim E. van Adrichem, Thomas L. C. Jansen

**Affiliations:** Zernike Institute for Advanced Materials, University of Groningen, 9747 AG Groningen, The Netherlands

## Abstract

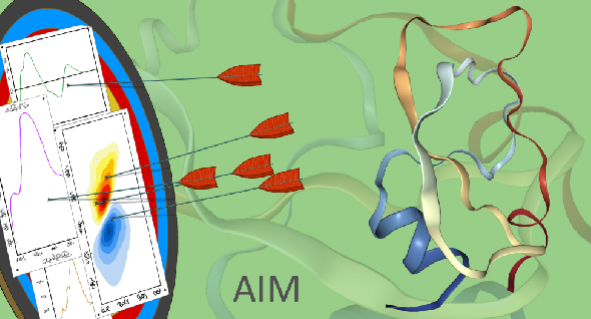

Here, we present a new analysis program, AIM, that allows extracting the vibrational amide-I Hamiltonian using molecular dynamics trajectories for protein infrared spectroscopy modeling. The constructed Hamiltonians can be used as input for spectral calculations allowing the calculation of infrared absorption spectra, vibrational circular dichroism, and two-dimensional infrared spectra. These spectroscopies allow the study of the structure and dynamics of proteins. We will explain the essence of how AIM works and give examples of the information and spectra that can be obtained with the program using the Trypsin Inhibitor as an example. AIM is freely available from GitHub, and the package contains a demonstration allowing easy introduction to the use of the program.

## 1 Introduction

Knowledge about the structure and dynamics of proteins is of great importance to understand their function.^1−3^ X-ray crystallography,^4^ nuclear magnetic resonance (NMR) spectroscopy,^5^ and cryogenic electron microscopy (cryo-EM)^6^ are powerful techniques to address protein structure. However, these methods are not well suited for obtaining the structure of all proteins, and in particular, information about protein dynamics, therefore alternatives are needed. Infrared (IR) spectroscopy^7,8^ often represents such an alternative due to its time resolution. However, this method does not have the desired atomic resolution. Therefore, modeling is of crucial importance for the interpretation of the IR spectra. In this paper, we will report on the development of a new program that improves the current schemes for connecting information on structure and dynamics obtained from molecular dynamics (MD) simulations with spectroscopic observables, which can be calculated with spectral simulation software. Our focus will be on the protein amide-I band, which is known to reveal information on the protein secondary structure.^9−12^

**Figure 1:**
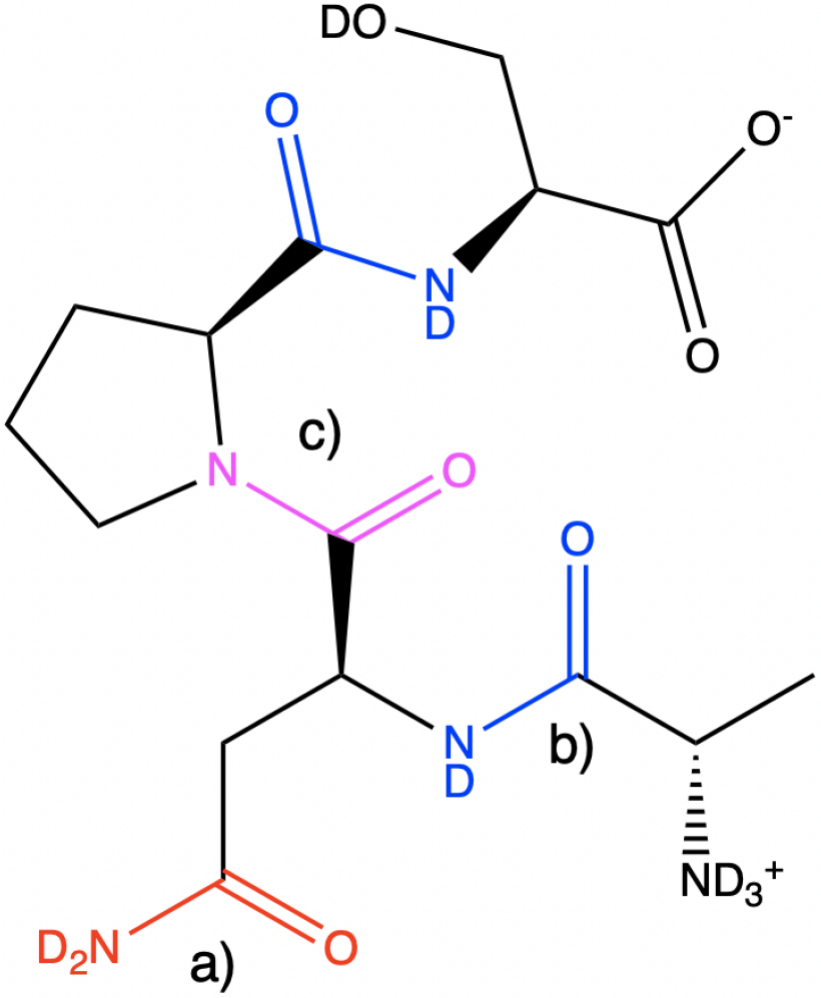
a) The primary amide found in the glutamine and asparagine side chains. b) Secondary amide found in the protein backbone. c) The tertiary amide group found at sites before proline in the backbone (also denoted preproline) units. The carbon, oxygen, nitrogen, and deuterium atoms involved in the amide-I vibration are highlighted with color.

The amide-I vibration is dominated by the CO-stretch in a peptide bond. In Figure 1, typical amide-I vibrations are illustrated in a small peptide. The absorption cross-section of the amide-I vibration is large as the highly polar CO-bond results in a large transition-dipole moment of the vibration. This also results in strong couplings between different local amide-I vibrations and delocalization of the normal modes, which are the observable vibrations in the spectroscopic experiments. The resulting collective nature of the amide-I vibrations makes this spectral region sensitive to the secondary structure and therefore popular for studying protein structure and dynamics.^8^

The bend vibration of water absorbs at ∼1650 cm^–1^, which overlaps with the amide-I spectral region. To ease interpretation of the spectra and limit spectral overlap, experiments are often performed in heavy water, which shifts the water bend vibration out of the spectral window to ∼1200 cm^–1^.^13^ This also results in (partial) exchange of the acidic hydrogen atoms in the protein with deuterium. This includes exchange of the hydrogen atoms involved in the amide-I vibrations, which shifts these vibrations by about 10 cm^–1^.^14,15^ To distinguish the new vibrations, they are often denoted amide-I′. Here, we will use the term amide-I to describe both, but unless otherwise stated explicitly, we will assume that the amide-I′ vibration is actually considered.

Gas-phase infrared spectra of peptides can be calculated efficiently using standard quantum chemical methods. However, for solvated proteins, such calculations become prohibitively computationally costly. This stems from three basic reasons. First, real proteins contain hundreds of atoms. Second, the solvent affects the vibrations and must be included explicitly. Finally, the systems are dynamic, and therefore, calculating the vibrational frequencies once does not suffice. For absorption spectra, a few hundred frequency calculations might suffice for the full protein, while for two-dimensional infrared spectra,^7^ thousands of frequency calculations are needed to account for the dynamic line shape. To overcome this challenge, the idea of frequency mappings emerged.^16^ Essentially, the vibrational frequencies are approximated using maps connecting them with the local electrostatic environment. Such maps are based on first-principles calculations or empirical fitting. Combined with spectral simulation methods^17^ this allows predicting the infrared spectra of entire proteins. This simulation protocol has successfully been applied in the study of protein structure and dynamics both using infrared absorption and two-dimensional infrared spectroscopy. The applications include the study of protein folding,^18−21^ the dimerization of insulin,^22,23^ the gating mechanism of the influenza M2 channel,^24^ the function of potassium channels,^25−27^ the formation of amyloid fibril structures,^28^ and the electrochemistry of cytochrome *c*.^29^ Computational spectroscopy has, thus, been demonstrated as a powerful tool in interpreting and predicting infrared spectra. However, general software, which is easy to use for nonexperts, is still not readily available.

The remainder of this paper is organized as follows. First, in the Methods section, the essential simulation protocol is summarized, and the role of the AIM program^30^ in this protocol is outlined. Furthermore, the advantages of AIM compared to existing mapping programs are described. This is followed by a section demonstrating examples of the application of AIM to both GROMACS and NAMD molecular dynamics trajectories. AIM is freely available for download on GitHub,^30^ and example files are included in the Supporting Information of this paper. Finally, the paper ends with the Conclusion, which includes an overview of our ideas for near future extensions of the program.

## 2 Methods

**Figure 2:**
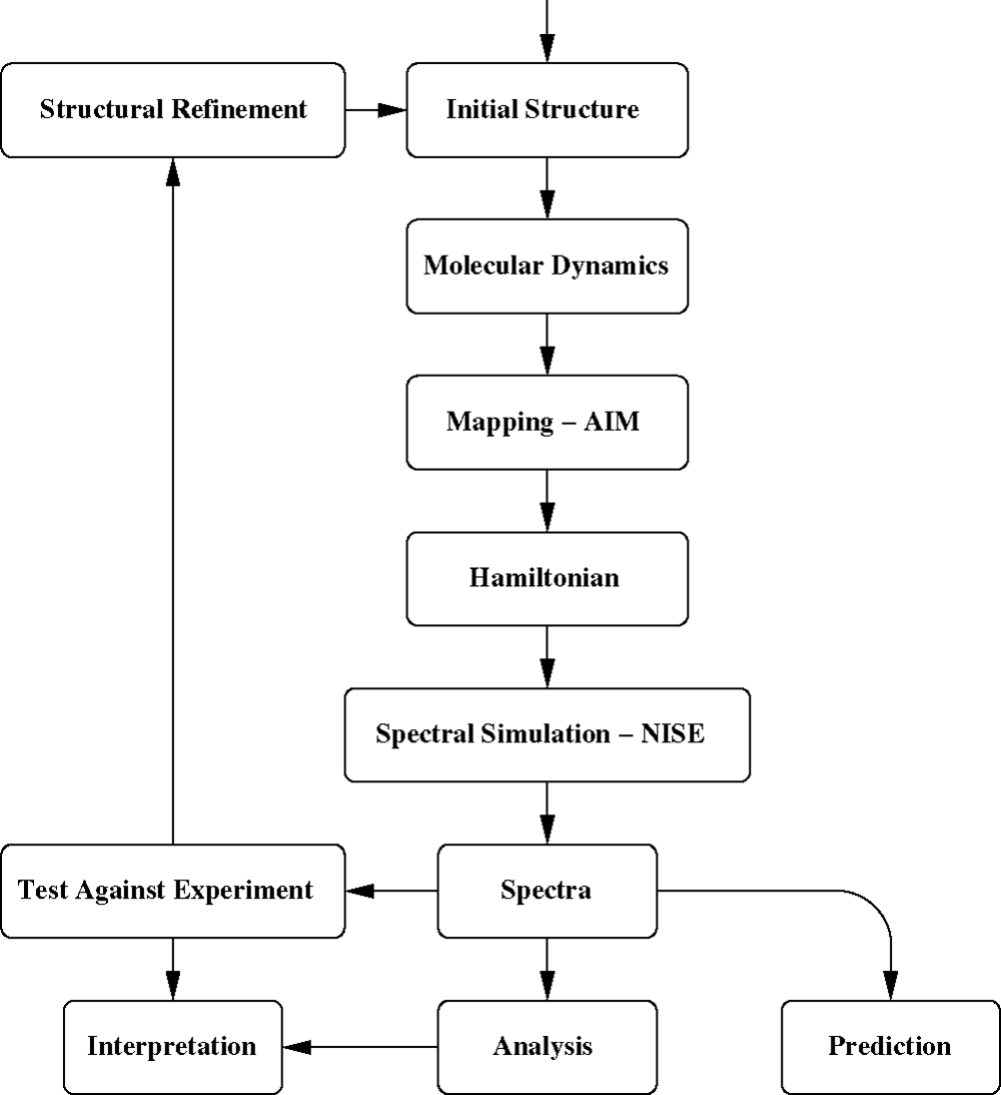
A schematic illustration of the workflow for calculating amide-I spectra.

The overall workflow for calculating amide-I spectra is illustrated in Figure 2. The AIM program takes a central place in this workflow. In essence, one will have to start by using an initial guess structure for the protein. This could, for example, come from the Protein Data Bank^31^ or structure prediction software such as AlphaFold.^32^ A molecular dynamics simulation is then performed to generate a representative structure and dynamics. This can involve sampling methods such as replica exchange^33^ or coarse-grained molecular dynamics coupling with backmapping schemes^34^ to produce atomistic trajectories. Atomistic molecular dynamics trajectories with dense sampling (10–20 fs between stored frames) are then used as input for the AIM program. AIM will generate a time-dependent Hamiltonian for the amide-I vibrations along with a trajectory of the transition dipoles and a trajectory of the center of the positions of the amide-I modes. This is the required input for the amide-I spectral calculations of linear absorption, linear dichroism, vibrational circular dichroism (VCD), isotope-label spectra, and two-dimensional infrared spectroscopic (2DIR) signals. AIM is designed to directly interface with the NISE^35^ program package, but the generated trajectories can also be converted to be used in the Spectron^36,37^ and g_spec^38^ packages. These programs also allow for the analysis of the origin of spectral features. Comparing the calculated spectra with experimental observations then allows spectral interpretation and potentially refinement of the structural model of the investigated protein. The following description will focus on the general properties of the Hamiltonian trajectories and the main options available in AIM to extract these Hamiltonians from molecular dynamics trajectories. For proteins of which the spectra are not yet known, the procedure can further be used to predict the spectra and for proposing ways of distinguishing different potential structures.^39^ A brief outline of the procedure used for spectral calculations in the NISE program will also be provided.

The amide-I vibrations are described with a vibrational Hamiltonian of the form^7^
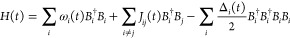
1where *i* labels each amide-I site, and *B*_*i*_^†^ and *B*_*i*_ are the Bosonic creation and annihilation operators, respectively. ω_*i*_(*t*) is the vibrational frequency of the local amide-I mode *i* at time *t*, *J*_*ij*_(*t*) is the coupling between two local modes, and Δ_*i*_(*t*) is the anharmonicity of the amide-I vibration on site *i*. The anharmonicity is needed for two-dimensional infrared spectra, where double excited states can be reached, as a result of two consecutive excitations. The time-dependence of the parameters in this Hamiltonian originates from the fluctuations of the protein structure and the local environment. Extensive research has been performed in the community to develop so-called frequency mappings to predict these parameters based on information about the local structure and environment.^16^ Below a more detailed discussion of the different aspects of such mappings will be outlined. The AIM program allows using the most popular of these mappings to create vibrational amide-I Hamiltonians from molecular dynamics trajectories of proteins.

The local amide-I vibration is typically described with a mapping that depends on the local electrostatic potential, field, and/or gradient on the carbon, oxygen, nitrogen, and/or deuterium atom of the central amide group. Furthermore, a nearest neighbor frequency shift is sometimes added as described below. We have implemented the most common electrostatic maps in AIM.^40−44^ The package also allows for user-defined mappings which should be straightforward to add when they depend only on the parameters discussed above.

The simplest approach for extracting couplings is using the transition-dipole coupling with the point-dipole approximation. The local amide-I modes are then considered vibrating point dipoles, and the coupling between pairs of vibrating point dipoles is given by the equation
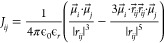
2Here, *r*_*ij*_ is the distance between the two dipoles,  and  are the transition dipoles, and ϵ_*r*_ is the relative dielectric constant. AIM also includes the more elaborate transition charge coupling scheme,^45−47^ which includes higher order multipole corrections to the interaction. The relative dielectric constant can be provided as an input parameter, scaling the long-range couplings depending on the (optical) dielectric environment. Previously, a value of ϵ_*r*_ = 1 gave reasonable results compared with experiment.^47−49^ A benchmark study^50^ suggests that setting ϵ_*r*_ = 1.5 may also be an appropriate choice.

As the electrostatic approximation may not be sufficient for short-range interactions, DFT calculations have been used to parametrize vibrational frequency shifts caused by the nearest neighbor groups along the protein backbone.^42^ These mappings also provide the couplings between the neighboring amide vibrations. In essence, the maps were created by calculating the amide-I modes for peptide dimers with DFT and varying the Ramachandran angles systematically in combination with a Hessian reconstruction procedure. The frequency shift of the two neighboring units and the coupling between the two can then be determined using bilinear interpolation on the grid of values determined with DFT.

The anharmonicity of the amide-I mode is typically set to 16 cm^–1^ as determined in early experiments.^7^ This value was also found in one mapping study.^42^ It was found that the anharmonicity does not fluctuate a great deal, and using a fixed value is often a reasonable approximation. The current version of AIM does not include a specific mapping of the anharmonicities, and a constant value is assumed.

A backbone amide unit located just before a proline unit will be a tertiary amide (see Figure 1c). This unit has a vibrational frequency that is typically about 27 cm^–1^ below that of the secondary amide units.^51^ Only the Jansen map was developed for this unit, and other maps implemented in AIM at this point apply a simple 27 cm^–1^ redshift for preproline groups to correct the frequency. We note that this value can be changed by the user, and it is possible to combine the preproline Jansen map with the other maps for the backbone in the program.

The side chains of glutamine and asparagine contain a primary amide-I unit (see Figure 1). The vibrational frequency of a primary amide is typically 30 cm^–1^ above that of a secondary amide.^41^ The Skinner map was developed with an explicit side-chain map, which is included in AIM. For all other map choices, the side-chain frequency is calculated with the secondary amide map and blue-shifted by 30 cm^–1^. We note that this value can be changed by the user, and it is possible to combine the side-chain Skinner map with the other maps for the backbone in the program.

Isotope edited vibrational spectroscopy is a powerful tool for revealing local structural and dynamical information. The formal frequency shift is given by the harmonic oscillator formula , where *k* is the force constant, and μ is the reduced mass. Assuming that only the reduced mass changes upon isotope editing, this results in a frequency scaling. In practice, a systematic frequency shift of about −41 cm^–1^ is observed for ^13^C labeling^21^ and −66 cm^–1^ for combined ^13^C^18^O labeling.^50^ Often vibrational spectroscopy for the amide-I band is performed in D_2_O to avoid interference with the H_2_O bend around 1650 cm^–1^.^52^ This leads to an exchange of hydrogen with deuterium of the acidic protons including that of the peptide bond.^53^ This changes the gas-phase frequency by 10 cm^–1^.^54^ It was demonstrated that this is neither well described by a simple shift nor with a simple change of reduced mass.^15^ Therefore, the spectra in D_2_O can better be modeled explicitly with mappings taking the deuteration explicitly into account as the Skinner,^41^ Jansen,^42^ and Tokmakoff^40^ ones implemented in AIM. Isotope edited spectroscopy with ^13^C and ^18^O labels can easily be performed as postprocessing of the Hamiltonian trajectory generated by AIM. Often the approximation that the isotope labeled units are uncoupled from the other units is further applied. This allows for efficient calculations by truncating the Hamiltonian to the labeled units only for the spectral calculations.^26^ Such truncated Hamiltonian can also be obtained directly with AIM.

The main function of AIM is to extract the parameters for the Hamiltonian trajectory described in eq 1. This is typically done with 20 fs timesteps between the stored MD frames, as this is needed to sample the frequency fluctuations, which are important for motional narrowing observed in FTIR spectra and lineshapes observed in 2DIR spectra. AIM further allows storing a file with positions related to the vibrations. As a default, this is chosen to be the position of the carbon atom in the peptide bond. This, for example, allows calculation not only for the VCD spectra^12,55^ but also for various analysis purposes, where the knowledge of the position of the different sites is important.

The predecessor of AIM, AmideImaps,^56^ has many of the functionalities of this new program. However, that was a tool developed specifically for GROMACS^57^ and only worked for that specific package with support for GROMACS versions 3 and 4. AIM utilizes the MDAnalysis^58,59^ package and, in principle, allows the use of trajectories of all the MD-software packages that MDAnalysis can analyze. We have at this point implemented and tested the use of GROMACS,^57^ CHARMM,^60^ NAMD,^61^ and AMBER^62^ trajectories. This is also an advantage over other spectroscopic mapping programs such as g_amide.^63,64^ Other advantages of AIM include that protein recognition has been implemented to automatically identify and treat diverse structures including cyclic proteins and multichain systems. For most systems, this means that the program automatically detects all relevant chromophores and assigns the proper connectivity for the application of nearest neighbor mappings. Preproline residues are identified, and in the case of consecutive proline residues, all preproline units are treated as such. The program allows users to add mappings for nonstandard residues. It has been optimized and is about twice as fast as the previous AmideImaps when the optional precompiled c-libraries are used. This speedup is partially thanks to the implementation of a neighbor searching algorithm.^65^

## 3 Examples for the Trypsin Inhibitor

**Figure 3:**
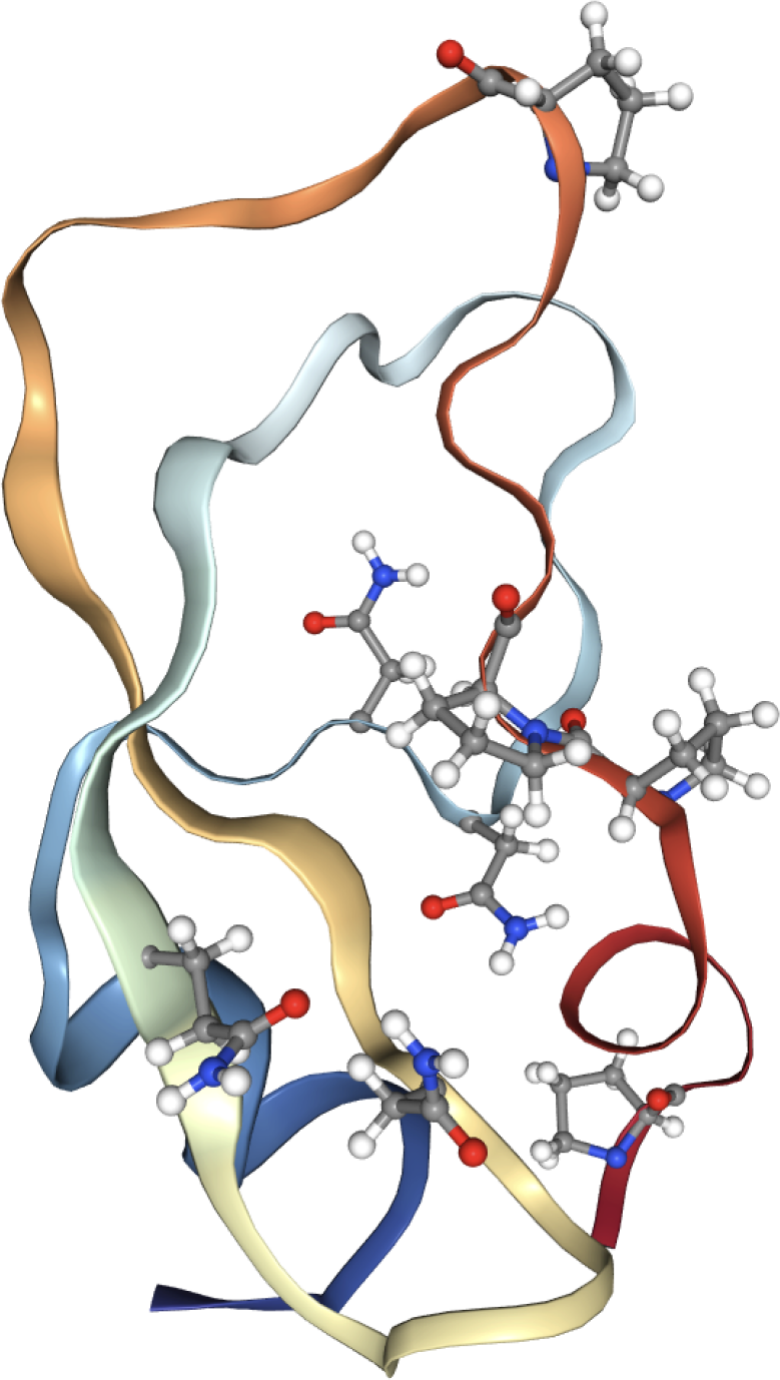
Structure of the Trypsin Inhibitor plotted using NGLview. The highlighted amino acids are the proline (Pro2, Pro8, Pro9, and Pro13), asparagine (Asn24, Asn43, and Ans44), and glutamine (Gln31) residues.^67^ The N-terminal is near the 3^10^-helix colored in red, and the C-terminal is near the α-helix colored in blue.

In the following, we will demonstrate the capability of the AIM program by the application to the Trypsin Inhibitor. This protein was chosen because experimental spectra are available for the same species as the Protein Data Bank structure and because it contains both an α-helix, β-sheet, and a short 3^10^-helix segment. The initial structure was taken from the Protein Data Bank^31^ using the 4PTI entry.^66^ The structure is illustrated in Figure 3. In the following, we will first describe the MD simulations performed with GROMACS and NAMD, then we will present the Hamiltonians obtained with AIM, and finally, we will present the resulting spectra obtained with NISE.

### 3.1 Molecular Dynamics Simulations

The GROMACS simulation structure was prepared by solvating the protein with SPC/E water^68^ covering the protein with a layer of at least 1 nm of water using periodic boundary conditions. A total of 6898 water molecules were included. Six chlorine counterions were added to keep the simulation box neutral. The protein and chlorine were treated using the OPLS-AA force field.^69^ The system preparation and simulations were performed with GROMACS version 4.6.3.^70^ Following an initial equilibration, the system was simulated for 1 ns using 2 fs time steps and saving every tenth frame. A 1 nm cutoff was used for the short-range interactions, and Particle-Mesh-Ewald^71^ was used for the long-range interactions. A simple 1 nm cutoff was used for the Lennard-Jones interactions. The temperature was kept constant at 300 K using the velocity rescaling scheme,^72^ and the pressure was kept constant at 1 bar using the Parrinello–Rahman barostat.^73^

The MD trajectory generated with GROMACS was analyzed using AIM. For all calculations, a cutoff of 2 nm was used for the electrostatic mappings,^74^ and the nearest neighbor coupling and frequency shifts^46^ were applied. The neighbor searching algorithm was applied with updates for every 50^*th*^ stored frame using a cutoff of 2.5 nm. The dielectric constant used for the long-range coupling was set to one. These settings replicate typical choices of previous studies.^49^ Two separate Hamiltonian trajectories were generated. For the first, we used the Jansen electrostatic map^42^ for frequencies and dipoles and the transition-dipole coupling scheme of Tasumi.^75^ For the other Hamiltonian trajectory, the Skinner electrostatic map was used^41^ together with transition dipoles of Torii^75^ and the transition-charge coupling scheme.^46^ In both cases, the position of the peptide bond carbon atom was stored as the position for the vibrational modes. These mapping combinations were chosen, as they were found to perform the best in the previous benchmark studies.^49^

For the NAMD simulations, the structure was prepared by solvating the protein with TIP3P water^76^ covering the protein with a layer of at least 1 nm of water using periodic boundary conditions. A total of 2547 water molecules were included. Six chlorine counterions were added to keep the simulation box neutral. The protein and chlorine were treated using the CHARMM36m force field.^77^ The system preparation and simulations were performed with NAMD version 2.14.^61^ Following an initial equilibration, the system was simulated for 1 ns using 2 fs time steps and saving every tenth frame. A 1 nm cutoff was used for the short-range interactions. and Particle-Mesh-Ewald^71^ was used for the long-range interactions. A 1 nm cutoff with a shifting function and a 0.9 nm shifting distance was used for the Lennard-Jones interactions. The temperature was kept constant at 300 K, and the pressure was kept constant at 1 bar using the Langevin algorithms for temperature and pressure.

The MD trajectory generated with NAMD was analyzed using AIM. For all calculations, a cutoff of 1.8 nm was used for the electrostatic mappings, and the nearest neighbor coupling and frequency shifts^46^ were applied. The slightly shorter cutoff was needed to avoid issues with the periodic boundary, as the simulation box was smaller than that of the GROMACS calculations. The neighbor searching algorithm was applied with updates for every 50^*th*^ stored frame using a cutoff of 2.5 nm. The dielectric constant used for the long-range coupling was set to one just as for the GROMACS trajectories. One Hamiltonian trajectory was generated. The Skinner electrostatic map was used^41^ together with transition dipoles of Torii^75^ and the transition-dipole coupling scheme of Tasumi.^75^

The reason for sampling the trajectories at 20 fs intervals is that the site energies are known to fluctuate on a sub-100 fs time scale^78^ leading to motional narrowing of the spectra. Furthermore, the used time step defines the spectral width available in the NISE algorithm.^79^ Previous benchmark studies found 20 fs to provide a good compromise between accuracy and computational cost.^48^ The use of thermostat and/or barostat may affect the dynamics in the molecular dynamics simulations and therefore the resulting spectra, which depend on the dynamics. Using the microcanonical ensemble or comparing results with and without a thermostat and barostat is, thus, recommended. For the present GROMACS simulations, this was tested, and no significant difference was observed on the spectra.

### 3.2 Hamiltonians

**Figure 4:**
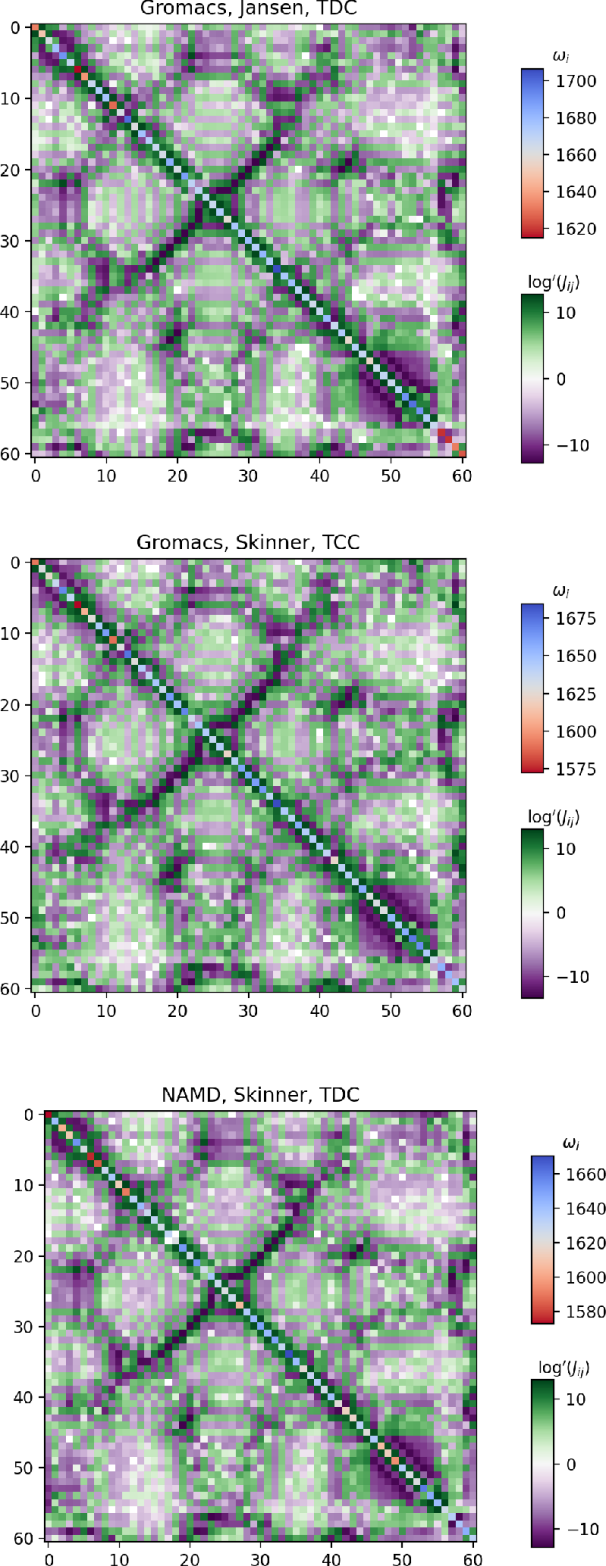
Comparison of the average of the two GROMACS based (top) and the NAMD based (bottom) Hamiltonian trajectories of the Trypsin Inhibitor. The indices 0 to 56 are the backbone residues. Indexes 57 through 60 are the side-chain units with Gln31 being index 58. The couplings are plotted using a logarithmic scale to enhance smaller values.

The average of each calculated Hamiltonian trajectory is illustrated in Figure 4. The pattern of the couplings reflects the main secondary structures. The 3^10^-helix from Asp3 to Leu6 is accompanied by strong negative neighbor couplings. The double-stranded antiparallel β-sheet from Ile18 to Tyr35 is visible through the antidiagonal line showing the cross-strand couplings where Lys26 is in the middle of the β-turn. The α-helix from Ala48 to Gly56 is connected with negative couplings between the involved units resulting from the largely aligned transition dipoles in the helix. The side-chain amides exhibit rather large couplings with each other as well as numerous backbone units.

### 3.3 Spectral Simulations

The spectral simulations were performed using the NISE_2017 spectral simulation package.^35^ For 2DIR simulations, couplings with a magnitude smaller than 0.01 cm^–1^ were neglected to speed up the calculations. The anharmonicity was set to 16 cm^–1^. The spectra were calculated in the spectral window from 1550 to 1750 cm^–1^ using coherence times from 0 to 2.56 ps in 20 fs increments. An exponential apodization function corresponding to an effective lifetime of 1.8 ps was used for spectral smoothening. The spectra were obtained by averaging over 1000 equidistant starting points along the trajectory. This follows commonly used simulation protocols.^12^

**Figure 5:**
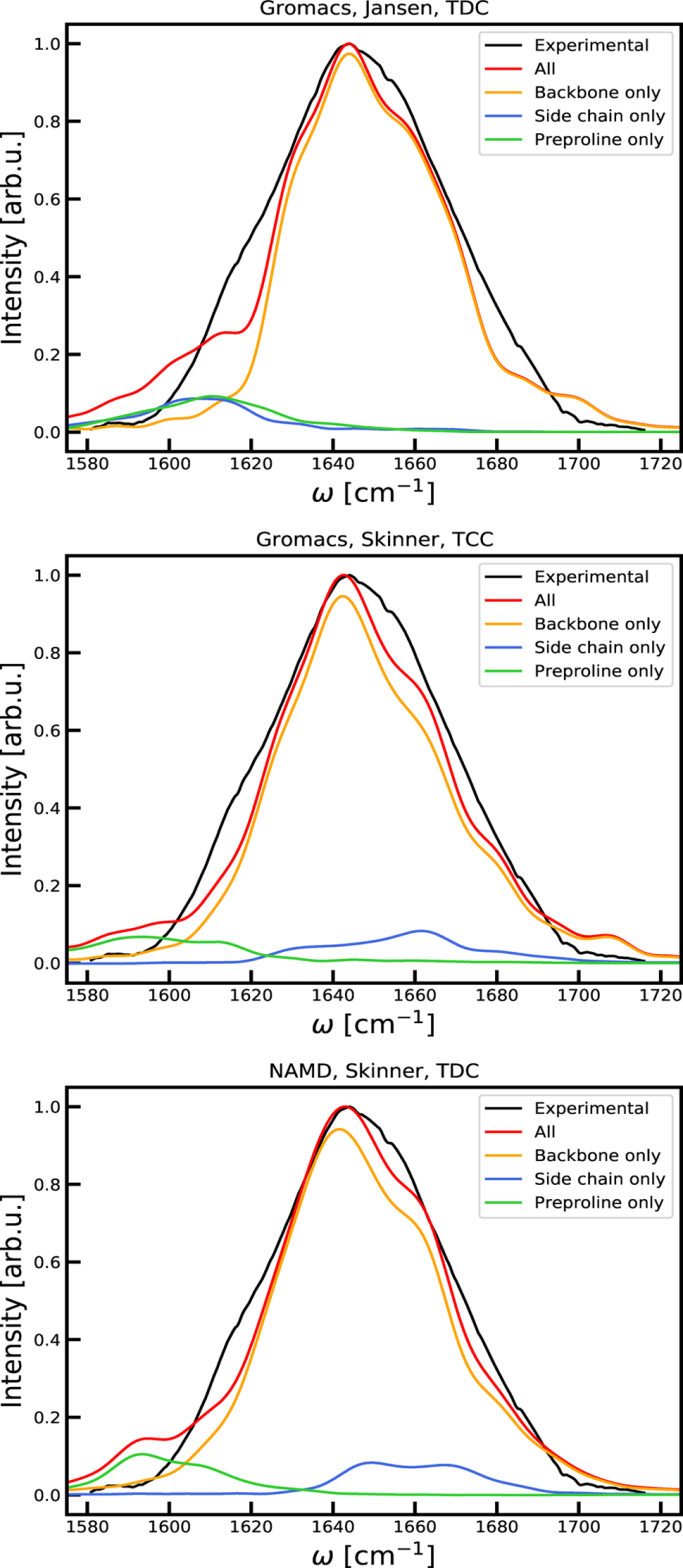
Predicted linear absorption spectra of the Trypsin Inhibitor. The experimental data are taken from ref (80). The frequencies of the simulated spectra were shifted to maximize the spectral overlap, as described in the text.

The linear absorption for all three simulations is shown in Figure 5 together with the experimental data from ref (80). A systematic peak shift was introduced by maximizing the spectral overlap with the experimental data as discussed in refs (48 and 49). The obtained shifts were −18.8, 7.2, and 21.0 cm^–1^ for the three trajectories which is comparable to the numbers reported in refs (48 and 49). The general line width and peak shape of the simulated spectra are in fairly good agreement with experiment. However, all the predicted spectra are slightly narrower than the experimental one. Subsystem spectra were calculated by projection on subsets of residues^29^ for interpretation. The projection on the preproline residues shows that these units predominantly contribute to the red part of the spectrum in all cases. The projection on the side-chain units gives very different results, when using the Skinner map specifically developed for these units and when using the shifted Jansen map. The shifted map underestimates the blue shift of the side-chain amides significantly. It is, thus, a better idea to use the Skinner map for side chains directly in combination with the Jansen map than to simply shift the Jansen map frequencies. This is in contrast to the preproline maps, where the shifted backbone Skinner map gives a reasonable result. More elaborate comparisons of different mappings are found in the literature.^47−49^

**Figure 6:**
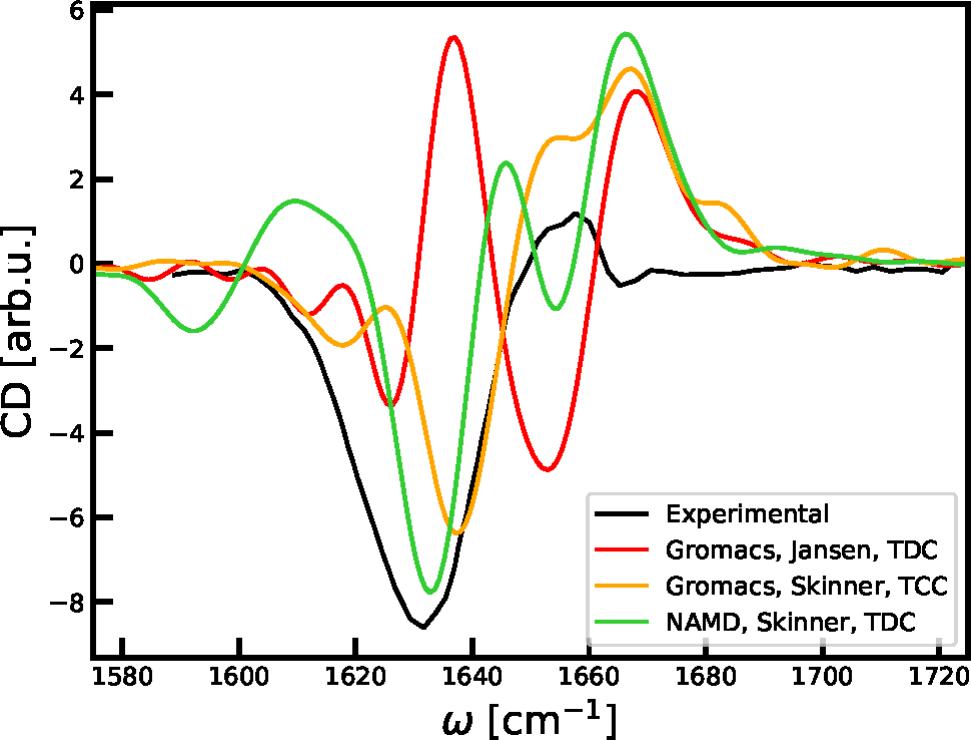
Predicted VCD spectra of the Trypsin Inhibitor. The experimental data was digitized from ref (82). The frequencies of the simulated spectra were shifted as for the absorption spectra.

The simulated VCD spectra^12,55,81^ are shown in Figure 6. For these simulations, the carbonyl carbon of the backbone was assumed to be the center of each site vibration, and intrinsic magnetic transition-dipole moments were neglected. The dependence on the simulation method is very clear, and the deviation from the experimentally reported spectrum^82^ is sizable. The used algorithm provides conservative circular dichroism spectra, while the experimental spectrum is predominantly negative. This can potentially be explained by mixing with the amide-II vibration, which is neglected here.^81,83^ It is generally known that circular dichroism is a very sensitive technique and, thus, challenging to predict. This, in principle, provides a handle for further improving the mappings in the future. Furthermore, the inclusion of coupling with other modes, the inclusion of magnetic dipole contributions, and the adjustment of the assigned positions of the transition dipoles may help improve the agreement in the future.

**Figure 7:**
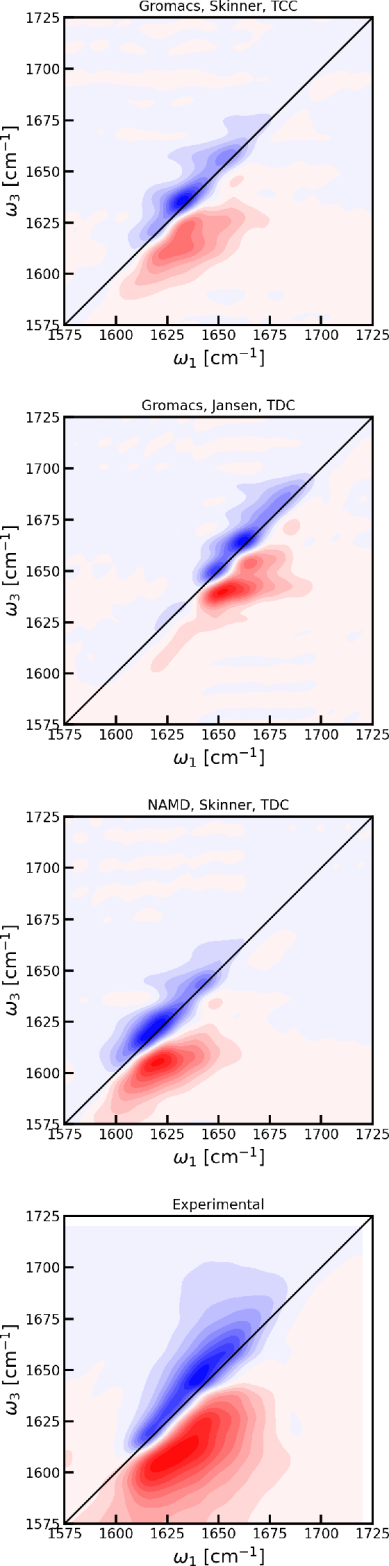
Predicted two-dimensional infrared spectra with zero waiting time and perpendicular laser polarization of the Trypsin Inhibitor. The contour lines are drawn for each 10% of the maximal signal. The red color indicates induced absorption, while the blue color indicates bleach. The experimental data are taken from ref (80).

**Figure 8:**
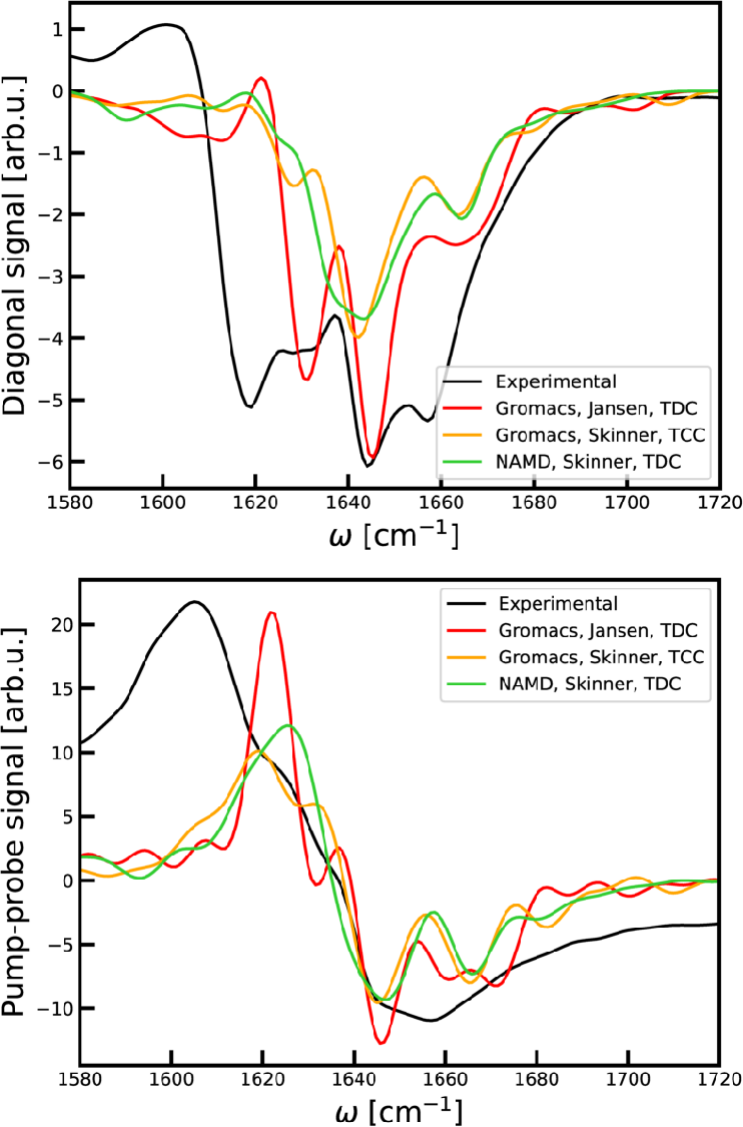
Top: Diagonal cuts through the 2DIR spectra of the Trypsin Inhibitor. Bottom: Broad-band pump–probe spectra of the Trypsin Inhibitor. The experimental data were obtained by analysis of data from ref (80). The frequencies of the simulated spectra were shifted as for the absorption spectra.

The two-dimensional infrared spectra obtained from the three trajectories described above are compared with experimental data^80^ in Figure 7. The frequencies in these spectra were not shifted. The predicted spectra are all narrower than the experimental spectrum. This is expected as the absorption spectra were already narrower. This means that the separate peak features stand out more in the simulated spectra. These features are quite similar in the three predicted spectra. Diagonal slices and broad-band pump–probe spectra are shown in Figure 8 to further highlight the differences between the spectra. The pump–probe spectra were obtained by integrating the 2DIR spectra over ω_1_. Overall, the NAMD trajectory seems slightly closer to the experimental result. The underestimation of the line width may be a result of either the amount of disorder in the system or an overestimation of the couplings. A potential source of undersampling of disorder may be the length of the trajectories, which were only 1 ns long here. Based on the calculations presented here and the previous benchmark studies already discussed, the mapping combination combining the Skinner backbone, the Skinner side chain, and the shifted Skinner map for the preproline units combined with the TCC coupling and the nearest neighbor corrections used in this study is made the default choice in the AIM program. Depending on, for example, the force field choice, the user may, of course, want to choose an alternative mapping combination.

The use of the NumPy^84^ and Numba^85^ libraries is important for the speed of the developed code. The time duration of the calculations described here will, of course, be highly system dependent. As an indication of the expected computational time requirement, the Skinner mapping calculations using the c-library took about 1 h on both a 2.3 GHz 8-Core Intel Core i9 under Mac OS and a 2.5 GHz (two Intel Xeon E5 2680v3 CPUs) with Linux. An absorption spectrum was calculated in about 12 s. A VCD spectrum took 10 min to calculate. Each 2DIR spectrum was obtained in 128 CPU h. The NAMD molecular dynamics simulations took about 2 CPU h. The AIM program is not running parallel, but it is easy to distribute subsets of a trajectory over different calculations. In the future, we intend to make a native parallel AIM version available via the GitHub site.^30^

## 4 Conclusion

We have described a protocol for simulating the amide-I band of proteins in solution. Our focus is on AIM, which is a new program developed to convert the structural and dynamic information obtained in molecular dynamics simulations to Hamiltonian trajectories that are used as input for spectral calculations. AIM is distributed freely as open-source software via GitHub.^30^ We demonstrated the application of the program to trajectories generated with GROMACS and NAMD. We discussed a few points that one needs to keep in mind for such calculations.

For the demonstration of AIM, we found the general coupling pattern to be very similar between models, while some variation was found between absorption spectra in accordance with previous studies.^47−49^ The variation between predicted VCD reflects the higher sensitivity to structural details of this technique. For the two-dimensional infrared spectra, we observed that they generally reproduce the same pattern, while the inhomogeneous line width was slightly underestimated in the simulations. The shift correction applied to preproline units for the Skinner map was seen to give sensible results, while a similar correction to the side-chain units for the Jansen map underestimates the blue shift predicted by the Skinner map. This suggests that the amide-I vibration of primary amides is quite different from that of secondary and tertiary amides.

The main advantages of AIM are that the program allows generating Hamiltonian trajectories from multiple molecular dynamics packages, it automatically identifies the different units in proteins, and it is about twice as fast as the predecessor. AIM in its current form is already a very powerful tool for the simulations of infrared spectra of proteins. However, we foresee a number of improvements in the future. One of these would be the inclusion of the calculation of transition polarizabilities needed for the simulation of Raman and sum-frequency generation signals. Verified interfacing with more MD packages will also be a useful addition. Furthermore, it should be easy to extend the code with more mappings, not only for the amide-I region but also for other vibrational modes. The code is already designed so that users can add these mappings independently.

## 5 Data and Software Availability

The data that support the findings of this study are available from the corresponding author upon reasonable request.

## Supporting Information Available

Description of zip file (PDF)
AIM_Examples.zip containing input for GROMACS and NAMD production run calculations and input files for AIM and NISE calculations (ZIP)
