# Supplementary material for: AIM: A Mapping Program for Infrared Spectroscopy of Proteins
Source: J Chem Theory Comput. 2022 Apr 7;18(5):3089–98. doi: 10.1021/acs.jctc.2c00113 (PMC9097285; doi:10.1021/acs.jctc.2c00113)
Supplement: Supplementary file 1 — ct2c00113_si_001.pdf [file ct2c00113_si_001.pdf]

Supporting Information for AIM: A mapping program for infrared spectroscopy of proteins

Kim E. van Adrichem* and Thomas L. C. Jansen*

University of Groningen, Zernike Institute for Advanced Materials, 9747 AG Groningen, The Netherlands.

E-mail: k.e.van.adrichem@rug.nl; t.l.c.jansen@rug.nl

A zip file named AIM_Examples.zip containing the input for the GROMACS, and NAMD production run calculations and the input files for the AIM and NISE calculations presented in this paper. The content of the included files and instructions to reproduce the calculations are provided in appropriate README files.
